# Transfer RNA and Origins of RNA Interference

**DOI:** 10.3389/fmolb.2021.708984

**Published:** 2021-07-23

**Authors:** Andrey Grigoriev

**Affiliations:** Department of Biology, Center for Computational and Integrative Biology, Rutgers University, Camden, NY, Uinted States

**Keywords:** tRNA, tRNA fragments, argonaute, microRNA, RISC, RNAi

## Introduction

Almost 30 years ago the first microRNA (miRNA) was detected (Lee et al., 1993), later put in mechanistical context by the discovery of the RNA interference (RNAi) and RNA-induced silencing complex (RISC). miRNA is the variable RISC element (guide) that recognizes messenger RNA (mRNA) targets in the RNAi process facilitated by Argonaute (Ago) proteins, the fixed key players in RISC. Alas, poetically sounding Argonautes were named not after the adventurers from Greek mythology but octopus-shaped leaves of an *Arabidopsis thaliana* mutant (Bohmert et al., 1998). Production of miRNAs is performed by a sophisticated orchestra of players with even groovier names, like Drosha, Pasha or Dicer. While roles of miRNA in post-transcriptional regulation have been established, its evolution has been typically described as expansion of new miRNA genes from the existing ones, not their first appearance (Berezikov, 2011). The emergence of RNAi has been attributed to its defense against pathogens, which involves other participants, e.g., double-stranded RNA binding proteins (dsRBPs). But details of such emergence are lacking.

This is a short opinion piece, not a full review, so I apologetically skip some of these players and hundreds of relevant miRNA/RNAi citations (and those for subsections below). The complexity of the RISC is bewildering. Intricate steps of miRNA production in the nucleus are followed by unequal strand loading to Ago, and finally resulting in cytoplasmic events, sometimes involving “slashing” of mRNA. All that (for simplicity, ignoring nuclear events upon Ago transport from cytoplasm) is based on weak and imperfect binding of short “seeds” (5′ 6-8 nucleotides in length in animals) to targets and on weak outcomes of slight tuning of target translation. How can such weak binding be a driving force for emergence and evolution of this complicated system and its parts, acting in concert in different cellular compartments?

Plant RNAi requires full-length small RNA hybridization, significantly limiting the number of target genes, yet the system appears even more complex (four Dicers, seven dsRBPs versus a single Dicer/couple of dsRBPs in humans). Further, the miRNA generation machineries are not entirely homologous, mature miRNAs are produced and modified in the plant nucleus, but these processes are divided between the nucleus and cytoplasm in animals. How would these different processes evolve in parallel with creating the elaborate and divergent Ago functionalities (four different Agos in humans, ten in *A. thaliana*, 26 in *Caenorhabditis elegans*), able to utilize these precisely cut miRNAs for mRNA regulation? This scenario would require a small RNA already acting together with some Ago prototypic protein. Compatible with this view, the last common ancestor of eukaryotes likely appeared after the RNAi functional principles (and some components, except for miRNA) had been invented (Cerutti and Casas-Mollano, 2006). And many prokaryotes do not have miRNAs but possess Ago protein homologs.

### Transfer RNAs and Their Fragments

Enter transfer RNAs (tRNAs), fundamental elements in mRNA translation. Each tRNA is a link of informational (anticodon) and corresponding chemical (aminoacid) units, which jointly provide the basis of the central dogma. These are ancient molecules, potentially capable of performing primitive replication (Kühnlein et al., 2021) or aiding in it (Maizels and Weiner, 1994). While the genetic code requires <64 anticodons for translation, tRNA genes are very numerous, with several hundred copies in human genome. From a regulation standpoint, these numbers far exceed potential codon adaptation mechanisms. Perhaps fitting to this view, many tRNA genes have been described as inactive, raising questions about their actual role (Torres, 2019).

The early sequence/structure determination and clearly defined function of tRNAs established their place in molecular biology textbooks, hardly revisited until the arrival of tRNA fragments (tRFs). Hypotheses of further roles were occasionally entertained, based on additional function observed, e.g., in viral replication, etc. (see also a review (Avcilar-Kucukgoze and Kashina, 2020) in this article collection for more details on these roles). However, an avalanche of data from small RNA sequencing experiments have challenged such perceptions, revealing numerous and ubiquitous tRFs in a multitude of sequenced samples. These fragments, detected in datasets produced to study miRNA, have been mostly dismissed as noise. It took >10 years to get acceptance even after detailed studies (Cole et al., 2009; Lee et al., 2009; Haussecker et al., 2010; Ivanov et al., 2011; Gebetsberger et al., 2012), even though tRNA breakage products were detected in urine of cancer patients much earlier (Speer et al., 1979). The finding of Ago proteins loaded with tRFs, in addition to their cargo of miRNAs, have prompted speculations about similar functionality of tRFs and search for their targets (Table 1).

**TABLE 1:** Representative studies on detection and analysis of Argonaute-loaded tRFs in different organisms.

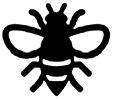	**Animals**
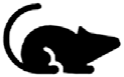	Cole et al. (2009), Haussecker et al. (2010), Burroughs et al. (2011), Li et al. (2012), Maute et al. (2013), Nie et al. (2013), Kumar et al. (2014), Karaiskos et al. (2015), Telonis et al. (2015), Hasler et al. (2016), Kuscu et al. (2018), Guan et al. (2020), and Guan et al. (2021)
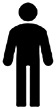
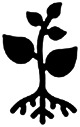	**Plants**
	Loss-Morais et al. (2013), Cognat et al. (2017), and Martinez et al. (2017)
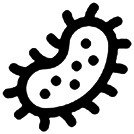	**Prokaryotes and unicellular eukaryotes**
	Couvillion et al. (2010), Olovnikov et al. (2013), and Garcia-Silva et al. (2014b)

By now, tRFs have been observed across all domains of life. There are multiple databases, containing different tRFs ever identified. For example, >28,000 tRFs are listed in MINTbase (Pliatsika et al., 2016), although the exact roles of most tRFs remain largely unknown, and only a dozen have been assigned a somewhat specific function. Yet, during the short period since their acceptance as *bona fide* cellular products and not sequencing noise, tRFs have provided several evolutionary surprises, exemplifying their regulatory effects not only within an organism but across time and space barriers.

***Time***: tRFs have been described as leading to metabolic disorders in the progeny of male mice on high protein or high fat diet (Chen et al., 2016; Sharma et al., 2016). This is an example of a non-Darwinian short-term trait propagation (to avoid the term “inheritance”) *via* incorporating tRFs into sperm. A bit like sending into the nearest future an information packet with father’s current environmental conditions. Did such signaling originate to get progeny better prepared for abundant food?

***Space***: a *Pseudomonas aeruginosa* vesicle-delivered tRF has been reported to affect the host immune response (Koeppen et al., 2016), while *Trypanosoma cruzi* tRFs—as contributing to susceptibility to infection of mammalian cells (Garcia-Silva et al., 2014a). Crossing species and kingdom barriers expands the idea of small RNA signals, e.g., the loading of tRFs into extracellular vesicles for likely cell-to-cell communication, such as T-cell de/activation (Chiou et al., 2018).

If regulation based on the weak binding of short RNAs to their targets is an ancient mechanism, we should see many more examples of intra-and interorganismal signals, exploiting this common principle. Notably, similar effects were reported in the classical RNAi papers on *C. elegans*. Progeny produced from the eggs of nematodes injected with dsRNAs showed interference phenotypes (Fire et al., 1998), while long dsRNA expressed in bacteria induced repression of genes with complementary sequences in worms fed with such bacteria (Timmons and Fire, 1998). Could these roles originate from tRFs (which apparently continue performing them, too)? If the tRF-driven regulation had led to the emergence of Ago functionality followed by the invention of RNAi, then one could imagine how divergent parts of plant and animal miRNA production arose based on this early mechanism.

### Turning a Weak Interaction Into a Strong Selective Force

The classical role of tRNAs co-exists with that of tRFs. What factors determine the fate of a given tRNA? And how does this relate to the origin of miRNA/RNAi?

A look at the evolutionary distant Archaea reveals some unusual but frequent tRNAs, potentially very relevant for dissecting the tRF function. These carry one or more introns, with the genomic order of tRNA parts occasionally permuted (Soma et al., 2007). Strikingly, units corresponding to tRNA exons sometimes also form 2 to 3 separate genes, as in the hyperthermophilic Archaea *Nanoarchaeum equitans* (Randau et al., 2005) or *Caldivirga maquilingensis* (Fujishima et al., 2009).

In protein-coding genes, exon borders are correlated with domain borders (Liu and Grigoriev, 2004), indicating how introns separate functional and evolutionarily mobile protein parts (Liu et al., 2005). This principle may also exist in tRNAs, although the introns were hypothesized to be mobile there (Fujishima et al., 2010). But what about the multi-gene tRNAs above? These unusual tRNAs may indicate separate functional roles of their parts, in addition to being involved in forming a whole tRNA from pieces. The split genes have been discussed in the context of tRNA origin (Di Giulio, 2008; Kanai, 2015). However, they also may represent traces/features of early tRFs. It would be interesting to check whether such exon units correspond to functional tRFs in these archaeal species. Notably, a map of intron insertion sites (Sugahara et al., 2009) shows they occur nearly everywhere between positions 12 and 60 on the canonical cloverleaf structure (encompassing the D- and T-loops). The majority of tRFs stored in the databases have ends in these positions—does this reflect sequencing noise, diversity of tRFs or potential ancient links with tRF production?

Such arrangements of tRNA exons quite possibly indicate additional functions of these exons, especially when they separate so much as to form individual sub-tRNA genes. And not just of the exons but of broken/digested tRNA pieces. Or, at least, this may be how all this had started, with random fragments present in the right place at the right time.

Role of tRNAs in translation makes their fragments ideal candidates for regulating this fundamental process. Proximity (essentially a macro-term for elevated concentration) is a very significant factor in evolution. For example, several ribosomes translating the same mRNA in bacteria produce identical proteins. These proteins are always formed close to each other, hence any mutation that carried even a small fitness advantage from enhancing their homotypic interactions would likely be selected for (compared to proteins separated in space and time). This had possibly led to the formation of homodimers and higher-order homo-multimers seen in a very large number of modern proteins (Grigoriev, 2001).

Similarly, a constant presence of fragments of tRNA right next to translated mRNAs could turn any slight fitness advantage from interactions between these molecules into a strong evolutionary force, despite interactions themselves being relatively weak. A similar force could then further select RNA-binding proteins for Ago-like functionality of enhancing the tRF-mRNA recognition to form a first prototypic RISC. The theoretical multitude of possible primordial tRF-target interactions suggests that many of them probably had not followed the rules that would later involve Ago. And many still do not: LeuCAG tRF seems to unwind the helices of a target mRNA to enable its translation (Kim et al., 2017) in Ago-less manner. Is this process catalyzed by another protein? Possibly. The very existence of these interactions could give rise to positive selection of other proteins for such catalysts (as with Ago, enabling more targeted tRF binding).

After a rudimentary RISC with this Ago-like functionality on tRFs was invented, a simple change would allow it to accept other small RNAs. After all, current Agos are very promiscuous RNA binders, “inviting” other short RNAs. While tRNA have constraints imposed on the sequence by their structure and essential function, candidate miRNAs may have seemingly any sequence, as long as it produces a recognizable hairpin for Drosha, Dicer & Co.

Ubiquitous small hairpins in the transcribed parts of a genome (from existing introns or appearing *de novo*), appropriately cut to provide a short RNA, could greatly enrich the repertoire of regulatory guides (and targets they regulate). That would drive the birth to thousands of miRNAs (and notably, some of miRNAs have recently been recognized as tRFs). Given the reports on likely roles of tRFs in regulating some of the more recent inventions in organism capabilities, such as neuronal or immunological, tRFs may be outnumbered by relatively unconstrained miRNAs but unlikely to be replaced.

Finally, I briefly revisit the emergence of RNAi for anti-pathogen defense. Although an attractive explanation for the RNAi existence, such defense seems unlikely to appear on its own, without a slashing agent, like Ago. How would a short RNA matching an arbitrary pathogen sequence “defend” against it without this protein? Perhaps by interfering with pathogen’s translation? This would again suggest that tRNA breakage products, abundant near translating ribosomes, might have been potential drivers of such interference. Then the defensive functionality of RNAi and its ability to regulate own mRNAs could have emerged almost simultaneously, following the same principles, and there is no need to develop defense as a predecessor of regulation.

In summary, while divergent modes of action seem to exist for tRFs, the outlined evolutionary path for an emergence of Argonaute-mediated gene regulation appears plausible.
